# Multimodal imaging of nano-assembled microspheres loaded with doxorubicin and Cisplatin for liver tumor therapy

**DOI:** 10.3389/fbioe.2022.1024174

**Published:** 2022-09-23

**Authors:** Yiwei He, Yuqing Zhang, Yuanchuan Gong, Zhewei Zhang, Tiancheng Xu, Liqiang Tian, Ting Pan, Hong Yang, Hao Pan, Quanming Kou, Hao Wang, Guoliang Shao

**Affiliations:** ^1^ Department of Radiology, Cancer Hospital of the University of Chinese Academy of Sciences (Zhejiang Cancer Hospital), Institute of Basic Medicine and Cancer (IBMC), Chinese Academy of Sciences, Hangzhou, China; ^2^ Second Clinical Medical College of Zhejiang Chinese Medical University, Hangzhou, China; ^3^ School of Automation, Hangzhou Dianzi University, Hangzhou, China; ^4^ Department of Neurosurgery, Linyi People’s Hospital, Linyi, China

**Keywords:** drug-loaded microsphere, embolization, multimodal imaging, DOX, Cisplatin, liver tumor

## Abstract

Currently, clinically available drug-loaded embolic microspheres have some shortcomings, such as being invisible with standard medical imaging modalities and only being able to carry positively charged drugs. The visualization of drug-loaded microspheres is very important for real-time monitoring of embolic position to improve the therapeutic effect. Meanwhile, the visualization of microspheres can enable postoperative reexamination, which is helpful for evaluating the embolization area and guiding the subsequent treatment. In addition, microspheres capable of loading different charged drugs can increase the choice of chemotherapeutic drugs and provide more possibilities for treatment. Therefore, it is of great importance to explore drug-loaded microspheres capable of multimodal imaging and loading drugs with different charges for transarterial chemoembolization (TACE) treatment of liver tumors. In our study, we designed a kind of nano-assembled microspheres (NAMs) that can realize computer X-ray tomography (CT)/magnetic resonance imaging (MRI)/Raman multimodal imaging, be loaded with positively and negatively charged drugs and test their imaging ability, drug loading and biological safety. The microspheres have strong attenuation performance for CT, high T_2_ relaxation for MRI and good sensitivity for surface enhanced Raman spectroscopy (SERS). At the same time, our microspheres can also load the positively charged drug, doxorubicin (DOX), and negatively charged drug Cisplatin. One gram of NAMs can hold 168 mg DOX or 126 mg Cisplatin, which has good drug loading and sustained-release capacity. Cell experiments also showed that the nano-assembled microspheres had good biocompatibility. Therefore, as multimodal developed drug loaded microspheres, nano assembled microspheres have great potential in TACE treatment of liver cancer.

## Introduction

Primary liver cancer is the sixth most commonly diagnosed cancer and the third leading cause of cancer-related death world-wide (Kishore et al., 2017; Sung et al., 2021). Due to the occult onset of liver cancer, most patients are already in the intermediate-advanced stage when diagnosed, and are not suitable for surgical resection due to the limitation of tumor size, number and metastasis. Transarterial chemoembolization (TACE) is an essential interventional treatment that selectively embolizes the tumor supplying artery by injecting embolic material and chemotherapeutic agents into the lesion. Moreover, TACE is recommended as the first-line treatment for intermediate-advanced stage hepatocellular carcinoma (HCC) by various guidelines such as the Barcelona Clinic Liver Cancer staging system (BCLC) and European Association for the Study of the Liver (EASL) (European Association For The Study Of The Liver. and European Organisation For Research And Treatment Of Cancer, 2012). Traditional iodized oil (lipiodol) embolization has been used for more than 20 years, but its clinical application has been limited by the difficulty of the mixed emulsion of lipiodol and chemotherapy drugs to exert stable and sustained anti-cancer effects, as well as serious systemic toxicity. In contrast, drug-eluting beads (DEBs) embolization maintains a high level of drug in the tumor areas for a long time, while reducing the drug concentration in circulation and mitigating systemic toxic side effects. Several clinical papers have reported that DEBs embolization is more effective than conventional TACE (cTACE) for hepatocellular carcinoma or hepatic metastatic tumors (Chen et al., 2017; Liu et al., 2018). However, TACE, based on DEBs, still has major shortcomings and faces challenges in development: 1) the DEBs cannot be visualized in real time, which prevents direct intraoperative monitoring of the DEBs for precise delivery and makes them prone to ectopic embolism; 2) the DEBs can only load positively charged drugs by electrostatic adsorption, which limits the choice of clinical chemotherapeutic drugs; 3) DEBs cannot be visualized by conventional imaging equipment, which prevents the acquisition of DEBs distribution information during postoperative review and affects subsequent treatment decisions (Sharma et al., 2010; Dreher et al., 2012; Ashrafi et al., 2017).

Therefore, the visualization of drug loaded microspheres and the search for new drug carriers have become a research hotspot (Sharma et al., 2016; Liu et al., 2021; Jia et al., 2022). At present, research on the visualization of microspheres mainly focuses on X-ray and magnetic resonance imaging (MR imaging). By introducing iodine, the interventionalist produced microspheres visible in X-rays that could be monitored during the injection and postoperative computed tomography (CT) scans (Sharma et al., 2010). MR imaging is mainly achieved by introducing paramagnetic lanthanides and superparamagnetic iron oxide into the microspheres (Fidelman et al., 2002). Some researchers have also studied CT/MRI dual-imaging microspheres with both CT and MRI internal contrast agents, which have been preliminarily verified in animal experiments (Hagit et al., 2010; Jia et al., 2022; Yin et al., 2022). Although previous studies have realized the visualization of drug loaded microspheres to a certain extent, they have not comprehensively solved the monitoring of drug loaded microspheres during injection, postoperative CT and MRI reexamination, or of distribution tracking. Therefore, the ideal drug-loaded microspheres should be capable of multi-modal imaging, so that intraoperative injection can be accurately monitored and the doctor can choose the appropriate imaging mode to trace the distribution of microspheres and the change of lesions according to their actual situation after surgery. At the same time, different charged drugs can be selected according to the actual efficacy. In addition, surface enhanced Raman spectroscopy (SERS) has attracted increased attention in tumor intraoperative imaging due to its high resolution and non-invasive advantages; thus, it may have great prospect in imaging navigation during embolization. Recently, some studies have reported that SERS imaging can be used to accurately delineate the margins of tumors intraoperatively and detect residual tumor, which significantly improves the effectiveness of the procedure and reduces the risk of disease recurrence (Jokerst et al., 2012; Andreou et al., 2016; Gao et al., 2017; Han et al., 2019; Shi et al., 2020). SERS can also be applied to build bioprobes to detect circulating tumor cells in peripheral blood, which is of great significance in early tumor diagnosis, efficacy evaluation, and postoperative condition monitoring (Xu et al., 2022). In the basic research of preclinical animal experiments for interventional embolization therapy, SERS imaging can precisely locate the position of microspheres in the blood vessels, which benefits the exploration of clinical treatment options.

**FIGURE 1:**
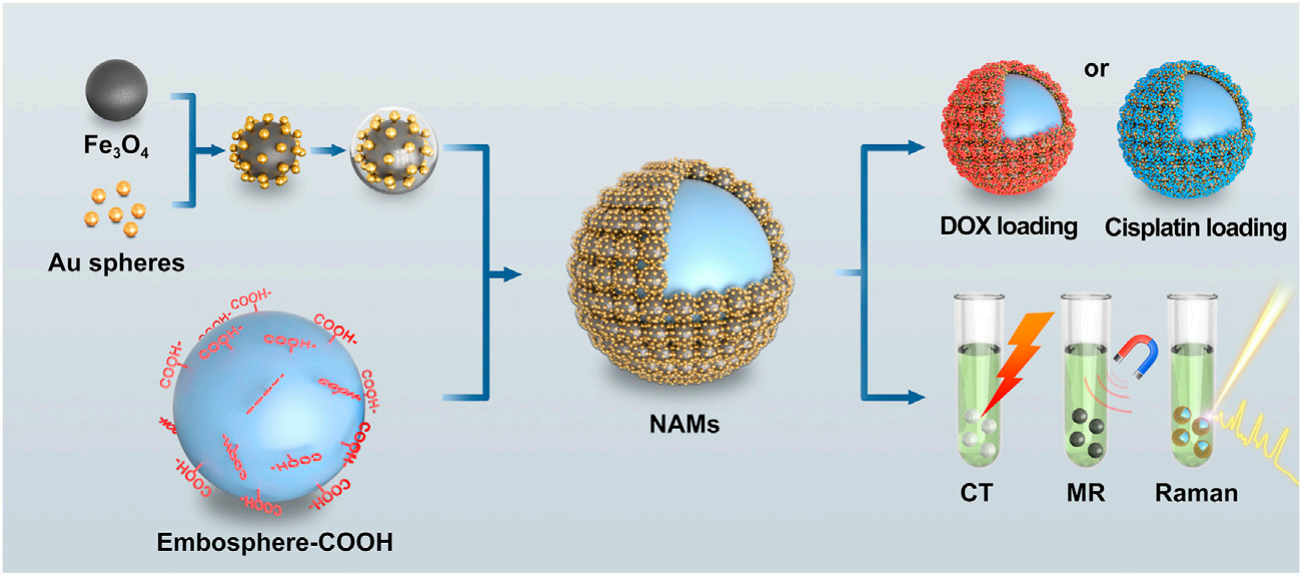
Schematic illustration of nano-assembled microspheres for multimodal imaging and drug loading.

In this study, we constructed a multimodal visualized nano-assembled drug-loaded microspheres, which can simultaneously realize CT/MRI/Raman visualization, vascular embolization, loading with positively and negatively charged chemotherapy drugs and expanding drug loading contents (as shown in Figure 1). With Fe_3_O_4_@Au nanoparticles as the assembly objects and Embospheres which are commonly used in clinical practice as the assembly subject, the assembly of nanoparticles and blank biological microspheres is realized through chemical bonding, and then the surface of the assembled microspheres is chemically modified to achieve a large drug loading content. One gram of NAMs can hold 168 mg of doxorubicin (DOX) or 126 mg of Cisplatin. Finally, the *in vitro* multimodal visualization ability, drug loading and release ability and biosafety were evaluated. The resulting nano-assembled microspheres have strong CT absorbance, enhanced MR imaging performance and a stable SERS signal. They can load chemotherapy drugs with positive and negative charges, and expand the drug loading capacity, which show great promise in TACE treatment of liver cancer.

## Materials and methods

### Materials

All materials were used as received, without further purification. Chloroauric chloride (HAuCl_4_·4H_2_O) and polyacrylic acid (PAA) were purchased from Sino-pharm Chemical Reagent Co., Ltd. (Shanghai, China). 4-Nitrothiophenol (4-NBT, 90%) was purchased from Fluorochem Ltd. (Derbyshire, United Kingdom). The cell counting kit-8 assay (CCK-8) was purchased from Dojindo Laboratories (Kumamoto, Japan). DMEM/high glucose medium, penicillin–streptomycin solution, fetalbovine serum (FBS), agarose and 0.25% trypsin-EDTA were purchased from Gibco Life Technologies (United States). Iron oxide (Fe_3_O_4_) nanoparticles, poly ethylene imine (PEI, 50%), Tris-HCl buffer, sodium polystyrene sulfonate (PSS), doxorubicin hydrochloride (98%), succinic anhydride, Cisplatin (99.95%) and dopamine hydrochloride (PDA, 98%) were obtained from Aladdin Chemical Reagent Co., Ltd. (Shanghai, China). Embospheres (100–300 μm) were purchased from Merit Medical (United States). Ultrapure water (UPW, 18.2 MΩ) was used for all experiments.

### Synthesis of small spheres -Fe_3_O_4_@Au@4-NBT@PDA nanoparticles

First, 30 nm Au spheres were synthesized using the sodium citrate reduction method (Jana et al., 2001). Then, 20 mg Fe_3_O_4_ nanoparticles (200 nm) were dissolved in 40 ml PEI solution (0.5 nM) and mechanically stirred for 3 h. The products were rinsed with water twice and collected using magnetic separation. Afterwards, the Fe_3_O_4_-PEI NPs were mixed with the as-prepared Au sphere solution (60 ml). After mechanical stirring for 4 h, the dissociated Au spheres were removed by magnetic separation and Fe_3_O_4_@Au NPs were dispersed in 40 ml UPW. Then 3 ml of 10 mM 4-NBT ethanol solution was added with shaking and the mixture was allowed to stand for 30 min. The supernatant was then removed by magnetic separation. The obtained Fe_3_O_4_@Au@4-NBT NPs were washed with UPW once and then dispersed in 30 ml Tris-HCl buffer. Subsequently, 3 ml PDA (26.4 nM) was added dropwise into the above solution, mechanically stirred for 3–5 h, and then the supernatant was removed by magnetic separation. The obtained Fe_3_O_4_@Au@4-NBT@PDA NPs (small spheres for brevity) were washed with UPW once and then dispersed in 30 ml UPW.

### Synthesis of nano-assembled microspheres

First, the surface of the Embospheres was modified with PAA. PAA (4 ml, 0.33 nM) was added dropwise into 1 ml Embospheres and stirred for 3 h. Subsequently, after washing twice with UPW, 50 ml small spheres were added and rotated for 30 min, and the nano-assembled microspheres were obtained. According to the different types of drug loading, we modified different groups on the surface of the nano-assembled microspheres. If DOX was needed, the nano-assembled microspheres were reacted with PSS to modify the sulfonic acid groups on the surface of the microspheres. If Cisplatin was needed, the nano-assembled microspheres were first reacted with dopamine hydrochloride and then reacted with succinic anhydride to modify the carboxyl groups on the surface of the microspheres.

### Characterization of nano-assembled microspheres

The morphologies of the as-synthesized nano-assembled microspheres were investigated on a JEM-2100 transmission electron microscope (TEM, Joel, Japan) and a Zeiss Sigma 300 scanning electron microscope (SEM, Zeiss, Germany). UV–vis extinction spectra were recorded on a Shimadzu UV-2600 spectrophotometer (Aucybest, Shanghai, China). The elemental composition of NPs was analyzed by inductively coupled plasma-mass spectroscopy (ICP-OES, Agilent, United States). Raman spectra were obtained on a confocal Raman system (Horiba, HR Evolution).

### Drug loading capacity and release studies (doxorubicin/Cisplatin)

To evaluate drug loading efficiency, 200 mg of DOX were dissolved in 16 ml UPW or 160 mg of Cisplatin in 16 ml UPW. Drug solutions were mixed with 1 g nano-assembled microspheres. DOX was loaded by gentle mechanical agitation at room temperature, while Cisplatin was loaded by gentle rotation at 45°C. The concentration of the supernatant was quantified at 0, 15, 30, 60, 90 min, and 24 h. The concentration of the DOX supernatant was quantified using a high performance liquid chromatography-mass spectrometry (HPLC-MS) system (Thermo Fisher UltiMate 3000). The chromatographic column used was a Hypersil Gold C18 (2.1 mm × 50 mm, 5 μm) from Thermo Scientific. A gradient program was applied with solvent A containing 0.1% formic acid in aqueous solution and solvent B containing 0.1% formic acid in acetonitrile. The flow rate was maintained at 0.2 ml/min. The injection volume was 20 μl. The mass spectrometer was employed in positive ionization mode. The concentration of Cisplatin supernatant was quantified by the ICP-OES spectrometer.

The loading capacity was calculated as follows: Loading capacity = initial concentration of drug-residual concentration in the supernatant. For the evaluation of elution, the above drug-loaded microsphere solutions were divided into two groups and placed in 120 ml of PBS at pH = 7.4 and pH = 5.6, respectively, and released by rotation at 37°C. Three replicates were evaluated and samples were collected at 15, 30 min, 1, 2, 4, 6, 8, 12, 18, 24, 48, 72, 96, 120, 144 and 168 h. The concentration of DOX was determined by the HPLC method and the concentration of Cisplatin supernatant was quantified by ICP-OES, as described above. The release rate of doxorubicin and Cisplatin was calculated at each time point.

### *In vitro* cytotoxicity

*In vitro* cytoviability assays were carried out using the cell counting Kit-8 (CCK-8) assay on human hepatocarcinoma Hep G2 cells. The cytoviability assay was divided into six groups: small spheres, nano-assembled microspheres, DOX, nano-assembled microspheres loaded with DOX, Cisplatin, and nano-assembled microspheres loaded with Cisplatin. Briefly, Hep G2 cells were seeded into a 96-well cell-culture plate at 10^5^/well in complete medium and allowed to grow at 37°C and 5% CO_2_ for 24 h. Then, five different grade concentrations (0.010, 0.019, 0.038, 0.076 and 0.152 nM) of the small spheres or (1,000, 2000, 5000,10000,20,000/ml) of the NAMs were added into the cells, which were treated in quintuplicate with fresh medium and incubated at 37°C under 5% CO_2_ for 24, 48 and 72 h, respectively. Cells treated with medium only served as a negative control group. The concentration gradient of DMEM solution for the other groups was set on this basis. 100 μl 10% CCK-8 DMEM solutions were added to each of the wells to replace the original medium and incubated for 2 h. Finally, cell viability was determined by measuring the light absorbance at 450 nm with a microplate reader (Thermo Multiskan Spectrum).

### *In vitro* multimodal imaging of nano-assembled microspheres

Solutions of nano-assembled microspheres at various concentrations were prepared in UPW in different 1.5 ml tubes for the phantom imaging test. The Raman signal measurement of nano-assembled microspheres with different concentrations were performed with 785 nm laser excitation, 1 s acquisition time/spectrum, and 100 mW laser power. The CT Hounsfield unit values of different elemental gold concentrations (5, 10, 15, 20, 40 and 60 mM) were measured using the SOMATOM Force CT (Siemens Healthineers, Forchheim Germany) with 120 kV, 150 mA, and a slice thickness of 0.5 mm. Images were reconstructed on the Syngo. *Via* VE 40B workstation (Siemens Healthineers, Forchheim Germany). CT values were acquired on the same workstation using the software supplied by the manufacturer. The contrast enhancement was determined via a linear fit of Hounsfield units (HU) for each sample. The small spheres with different iron concentrations were dispersed in 1.5% agarose solution. MR relaxivities of the as-synthesized nanoparticles were measured with a 3.0 T MR scanner (Philips 3.0T Ingenia CX). T_2_ calculate sequence was used to measure T_2_, and its parameters were TR = 2000 ms and TE = 10, 20, 30, 40, 50 and 60 ms, respectively.

## Results

### Characterization of nano-assembled microspheres

**FIGURE 2:**
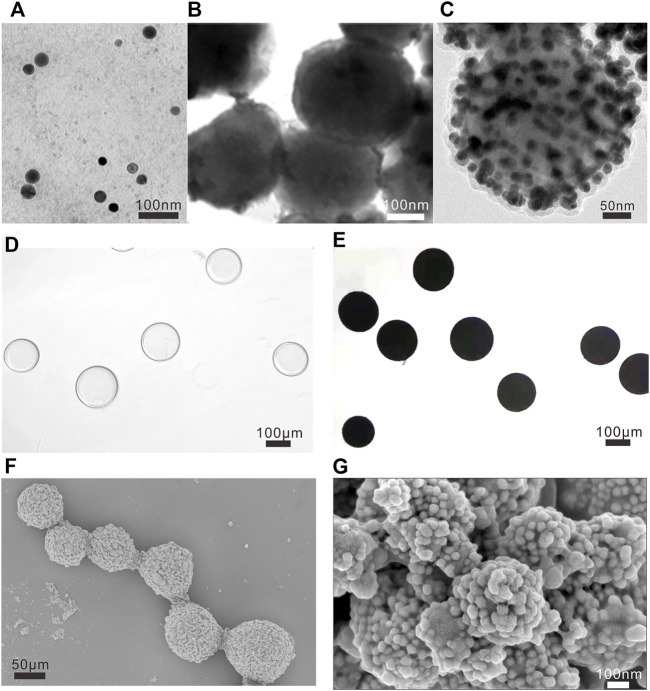
Characterization of NAMs. TEM images of **(A)** Au nanoparticles, **(B)** Fe_3_O_4_ nanoparticles and **(C)** small spheres, and the scalebar is 100 nm in **(A,B)**, and 50 nm in **(C)**. Morphologies of Embospheres before **(D)** and after **(E)** assembly with small spheres under optical microscope. The scale bar is 100 μm for **(D,E)**. Representative low-resolution SEM image [**(F)**, the scale bar is 50 μm] and high-resolution SEM images [**(G)**, the scale bar is 100 nm] of NAMs.

The synthetic process of the designed NAMs was characterized by various methods. The small spheres are composed of superparamagnetic iron oxides (SPIO), gold nanoparticles and Raman reporters of 4-NBT. In a typical experiment of preparing small spheres, 220 ± 13 nm uniform-sized Fe_3_O_4_ nanoparticles (Figure 2B) were modified with PEI and assembled with 30 ± 3 nm gold nanoparticles (Figure 2A) *via* covalent bonds, then 4-NBT molecules and dopamine were modified outside. Figure 2C shows a representative TEM image of small spheres with overall diameters of 310 ± 24 nm. Then, we assembled small spheres and PAA modified Embospheres resulting in the NAMs. Figures 2D,E show the morphologies of Embospheres before/after assembly with small spheres under an optical microscope. Before assembly, Embospheres were transparent microspheres; while after assembly, NAMs turned into black microspheres due to the color of the small spheres. SEM showed that the surface of the Embospheres became rough after assembly, and the morphology of the assembled small spheres on the surface can be clearly seen by local magnification. Even the 30 nm gold nanoparticles on the small spheres can be clearly seen (Figures 2F,G).

### Drug loading capacity and release studies (doxorubicin/Cisplatin)

Next, we selected two commonly used antitumor drugs (DOX with a positive charge and Cisplatin with a negative charge) to study the drug loading and release ability of NAMs. In this study, we did not use the traditional electrostatic adsorption drug loading method of clinical microspheres, such as Callispheres and DC beads (Ashrafi et al., 2017; Zhang et al., 2017), but used covalent bond binding for the drug loading. The sulfonate group on the NAMs surface was used to specifically bind DOX and the carboxyl group was used to bind Cisplatin for drug loading. For DOX loading, the red color of the DOX solution gradually faded while the NAMs gradually changed from black to red during the drug loading process. After DOX release, NAMs turns black again (Supplementary Figures S4A,B). Cisplatin is light yellow. The color of the NAMs did not change during the loading and release of cisplatin (Supplementary Figures S4C,D). The drug loading and release processes did not produce significant changes in the morphological size of the NAMs, and the structure of the NAMs was stable, nearly without dislodgement of the small spheres (Supplementary Figures S4E–G).

**FIGURE 3:**
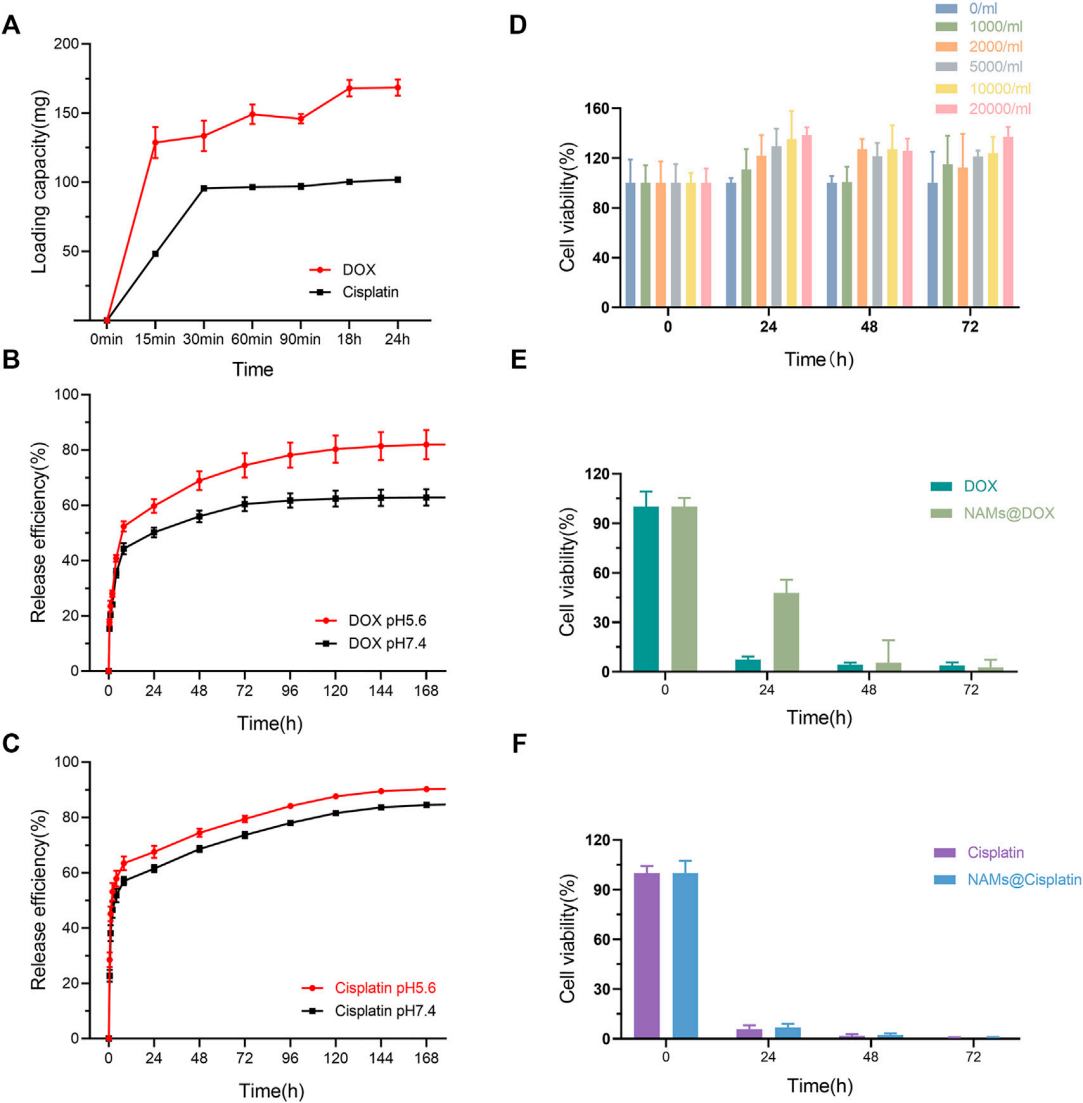
**(A)** Loading capacity of different type of drugs (DOX and Cisplatin); Release efficiency of DOX **(B)** and Cisplatin **(C)** in PBS with different pH (pH5.6 and pH7.4). **(D)** Cell viability of Hep G2 cells incubated with NAMs at different concentrations for 0, 24, 48, and 72 h. Data are shown as mean ± standard deviation (*n* = 6). Cell viability of Hep G2 cells treated with free DOX and DOX-loading NAMs **(E)**, and free Cisplatin and Cisplatin-loading NAMs, respectively **(F)**. Data are represented as mean ± standard deviation (*n* = 6).

As shown in Figure 3A, when the total amount of DOX added was 200 mg, 1 g NAMs can load 128 ± 11 mg of drug at 15 min, 149 ± 7 mg at 60 min, and then reached equilibrium. The loading capacity of NAMs at 24 h reached 168 mg for DOX, which was higher than that of Callispheres. Furthermore, the NAMs were able to load Cisplatin, which Callispheres cannot load. When the total amount of Cisplatin added was 160 mg, 1 g NAMs could load 117.3 ± 1.63 mg of drug at 30 min and then reached equilibrium. The loading capacity of NAMs at 24 h could reach 126 mg for Cisplatin.

Figures 3B,C showed the cumulative release percentage of NAMs loaded with different drugs in PBS at pH 5.6 and pH 7.4. Both DOX and Cisplatin released rapidly from the NAMs for the first 8 h and then slowed down. In pH 5.6 and pH 7.4 environments, the cumulative release efficiencies of NAMs were 84.65% and 59.39% for DOX, and 90.54% and 84.66% for Cisplatin within 168 h, respectively. And the result implied that, compared with Cisplatin, the pH of the release environments had a greater impact on the release efficiency of DOX, and the release of DOX was faster and higher in acidic environments.

### *In vitro* cytotoxicity

For biomedical interventional therapy applications, it is crucial to understand the cytotoxicity of the developed multifunctional NAMs. To quantitatively evaluate the cytotoxicity of NAMs and small spheres, we performed a CCK-8 assay to evaluate the cell viability of Hep G2 cells after incubation for 24, 48 and 72 h. The results showed negligible cytotoxicity of NAMs and small spheres when cocultured with Hep G2 cells, even after 72 h at concentrations up to 20,000/ml of NAMs or 0.152 nM small spheres (Figure 3D; Supplementary Figure S1). Unexpectedly, the growth of cells was enhanced at 24, 48 and 72 h, which is probably related to the large number of Au nano particles on the surface of NAMs which is close to the cells. However, the exact mechanism by which the large Au nanoparticles aggregates promotes cell growth is still poorly understood (Cui et al., 2012). Due to the property of NAMs sustained release drug, cell viability of Hep G2 cells treated with DOX-loading NAMs decreased gradually and reached the same level as free DOX within 48 h (Figure 3E; Supplementary Figure S2). The cell viability of Hep G2 cells treated with free Cisplatin and Cisplatin-loading NAMs reached the same level within 24 h (Figure 3F; Supplementary Figure S3).

### *In vitro* multimodal imaging of nano-assembled microspheres

**FIGURE 4:**
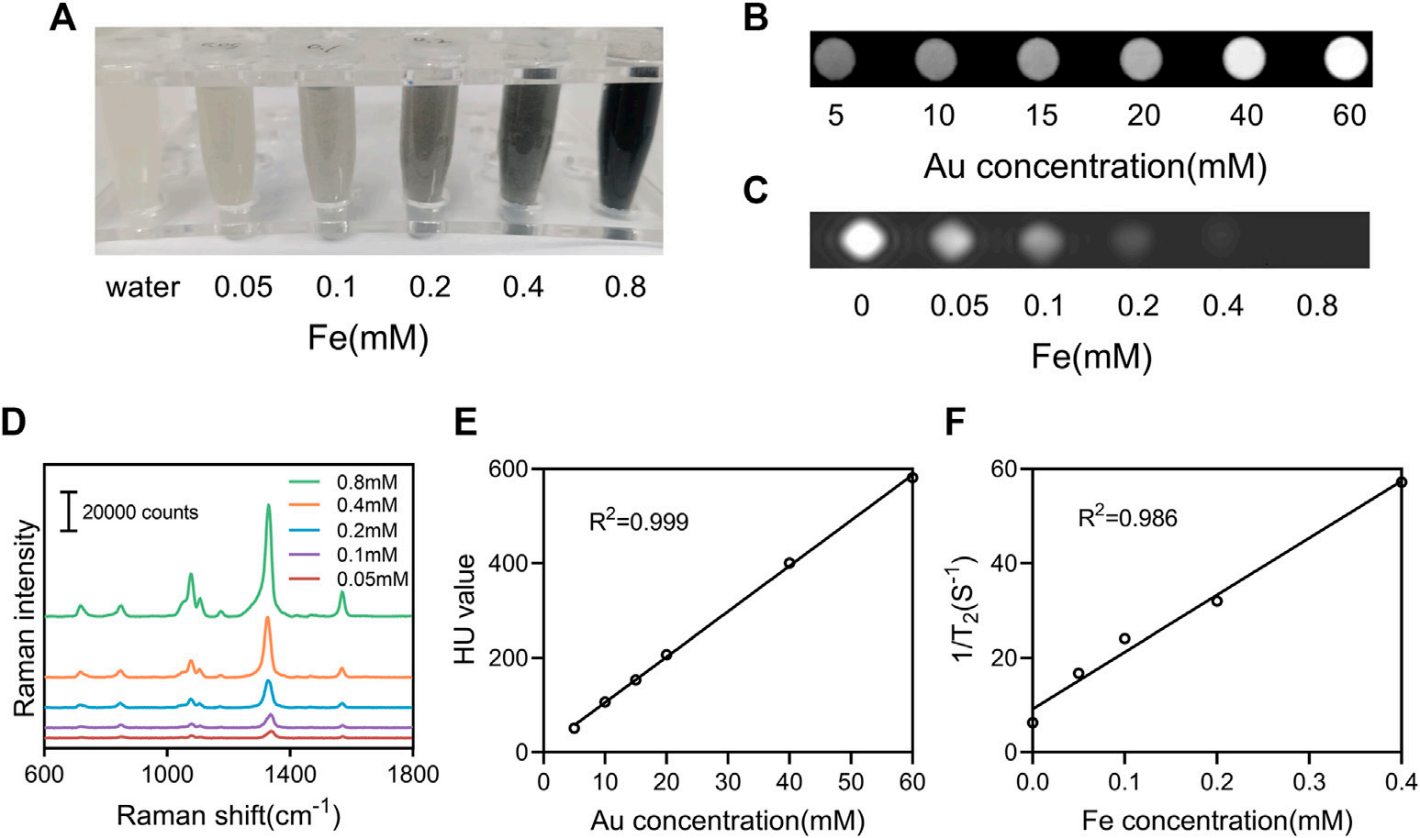
*In vitro* multimodal imaging of NAMs. **(A)** Photographs, **(B)** CT phantom images, **(C)** T_2_-weighted MRI phantom images, and **(D)** Raman spectra of the NAMs with various concentrations. **(E)** X-ray attenuation (HU value) of the NAMs at different Au concentrations. **(F)** The r2 relaxivity linear curve for the NAMs at different Fe concentrations.

With the coexistence of Au and Fe ions, they are expected to have both X-ray attenuation properties and T_2_ MR relaxivity. On the basis of the X-ray absorption coefficient rule, Au as one of high atomic number (Z) elements has been intensively utilized as the contrast agent for CT imaging applications. In our study, the increase in X-ray attenuation intensity as a function of Au concentration can be obviously verified by the observation of the CT images of the NAMs suspension with different concentrations. It can be seen that the brightness of the CT phantom increased with the number of NAMs increasing from 500 to 6,000 one tube, corresponding to an Au concentration from 5 to 60 mM (Figure 4B). Fe_3_O_4_-based materials are known to be good candidates for T_2_ -weighted MRI contrast agents. It can be seen that the NAMs were able to exert a positive contrast enhancement in a dose-dependent manner. The signal performance increased with increasing Fe concentration from 0 to 0.8 mM (Figures 4A,C). Further quantitative evaluations showed that the CT attenuation intensity (HU value) and T_2_ MR relaxivity both exhibited a linear dependence with the element concentrations (Figures 4E,F; Supplementary Table S1). In addition, the Raman signals increased gradually with increasing NAMs concentrations (Figure 4D). All these results confirmed the successful fabrication of the NAMs with strongly enhanced CT and T_2_-weighted MR imaging performances, as well as the highly sensitive SERS imaging capability. These properties enable NAMs to act as a new candidate for multimodal CT/MRI/SERS imaging applications.

## Discussion

TACE is the first-line treatment for advanced primary hepatocellular carcinoma, which maintains high chemotherapeutic drug concentrations in the tumor. Conventional TACE employs chemotherapeutic agents mixed with lipiodol to embolize the tumor supply arteries, which has serious systematic toxic effects (Gao et al., 2013; Vogl and Lahrsow, 2022). TACE based on drug-eluting microspheres also presents some challenges in the clinic. First, the drug-eluting beads cannot be visualized in real time, which prevents direct intraoperative monitoring, and thus, precise delivery of the microspheres and leaves them prone to ectopic embolism. Second, drug-loading beads carry drugs by electrostatic adsorption and can only load positively-charged drugs, which limits the choice of clinical chemotherapy drugs. In addition, the drug-loaded microspheres cannot be visualized by conventional imaging equipment, which prevents the acquisition of microsphere distribution information during postoperative review and affects subsequent treatment decisions (Sharma et al., 2010; Dreher et al., 2012; Ashrafi et al., 2017).

In our study, we prepared a novel type of nano-assembled microsphere, which synthesized multimodal visualized microspheres from SPIO, gold nanoparticles and Raman reporter 4-NBT, and assembled them with common clinical Embospheres. SPIO-based materials are known to be good candidates for T_2_ -weighted MRI contrast agents. Gold nanoparticles have been widely used for CT imaging given a high X-ray absorption coefficient and good biocompatibility compared with iodine-based contrast agents (Lee et al., 2013; Lusic and Grinstaff, 2013; Shi et al., 2020). The Raman reporter, 4-NBT, has a large scattering cross section, which can provide a strong SERS sensitivity (Zhang et al., 2019; Shi et al., 2020). The synthesized NAMs were able to achieve multimodal visualization in MRI/CT/Raman. MR and CT imaging are the most important modalities for staging and follow-up imaging of hepatocellular carcinoma (HCC) following catheter directed chemotherapy (Choi et al., 2014). The advantages of CT are that the modality is widely available, rapid, robust and requires less expertise to perform and interpret acquired images compared with MR imaging. The disadvantages include radiation exposure and relatively low soft-tissue contrast. MR imaging can provide superior soft-tissue contrast and permits the assessment of a greater number of functional tissue properties, which in principle should assist in lesion detection and characterization. However, given potential ambiguities during MR imaging (e.g., pre-existing signal voids prior to microsphere deposition), these MRI detection methods will likely require pre-infusion measurements, and thus, may be more time consuming and costly. Quantitative MRI methods typically require a greater level of expertise to perform and interpret images. Therefore, MRI and CT imaging are complementary for follow-up monitoring of tumor response following catheter directed interventions for the treatment of HCC. surface enhanced Raman spectroscopy (SERS) imaging has the features of being non-destructive, having high sensitivity, high specificity, high resolution and fingerprinting, etc. We labeled the NAMs with Raman signals, and SERS imaging can specifically identify the labeled microspheres, thus, providing high resolution and the precise location of microspheres in the tumor vasculature.

DOX and Cisplatin are commonly used as chemotherapeutic agents for the treatment of primary liver cancer. We loaded DOX by modifying PSS on the surface of NAMs. The loading capacity of DOX in NAMs was more than 80 g/L (168 mg per gram of Embospheres), which was higher than that in Callispheres with the same size of 100–300 μm (the maximum loading capacity was reported to be about 40 g/L in the literature) (Chen et al., 2021). The higher drug loading of NAMs may be due to the interaction of the sulfonic acid groups on the surface with DOX. Moreover, the NAMs can also carry Cisplatin, which cannot be carried by current clinical drug-loaded microspheres. The drug loading capacity of Cisplatin was up to 126 mg per gram of Embospheres, which greatly increased the choice of chemotherapeutic drugs in the clinic. The release of both DOX and Cisplatin was sustained for more than 1 week and had a continuous killing effect on tumors. Furthermore, the drug release efficiency was higher in the pH 5.6 environment, especially for DOX. The microenvironment of tumor tissues was exactly acidic, which is favorable for drug release.

## Conclusion

In summary, we have successfully developed nano-assembled microspheres for simultaneous CT/MRI/Raman visualization, vascular embolization, loading of positively and negatively charged chemotherapeutic agents, and expanded drug loading capacity. The synthesized NAMs demonstrated good stability, non-cytotoxicity and uniform size with strongly enhanced CT and MR imaging capability, and a high SERS signal. These multifunctional NAMs offer great potential for intraoperative real-time monitoring, increased chemotherapeutic drug selection as well as postoperative multimodal review. We believe the NAMs have great promise in improving d-TACE efficacy and reducing complications.

## Data Availability

The raw data supporting the conclusion of this article will be made available by the authors, without undue reservation.
