# Supplementary material for: Multimodal imaging of nano-assembled microspheres loaded with doxorubicin and Cisplatin for liver tumor therapy
Source: Front Bioeng Biotechnol. 2022 Sep 23;10:1024174. doi: 10.3389/fbioe.2022.1024174 (PMC9539659; doi:10.3389/fbioe.2022.1024174)
Supplement: Supplementary file 1 [file DataSheet1.docx]

Supplementary Material

# Supplementary Figures


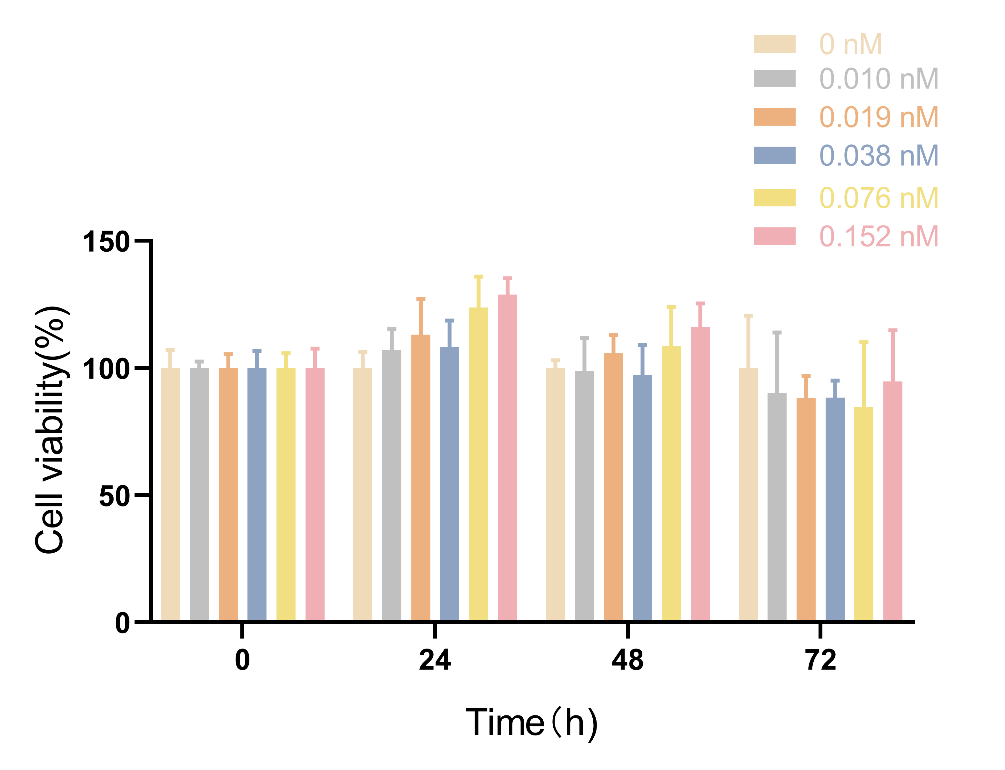


**Supplementary Figure 1.** Cell viability of Hep G2 cells incubated with small spheres at different concentrations for 0, 24, 48, and 72 h. Data are shown as mean ± standard deviation (n = 6).


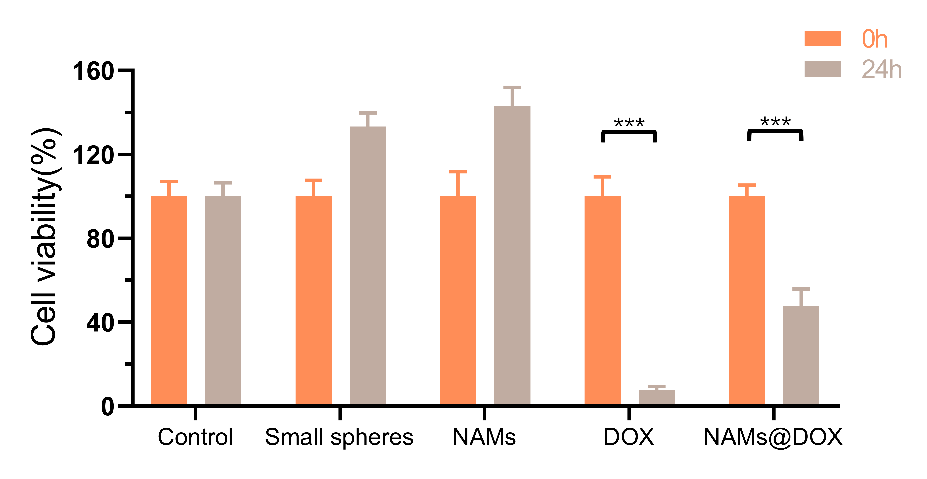


**Supplementary Figure 2.** Cell viability of Hep G2 cells treated with small spheres, NAMs, Free DOX and DOX-loading NAMs. Data are represented as mean ± standard deviation (n = 6). Significant differences are indicated by asterisks (***p < 0.001).


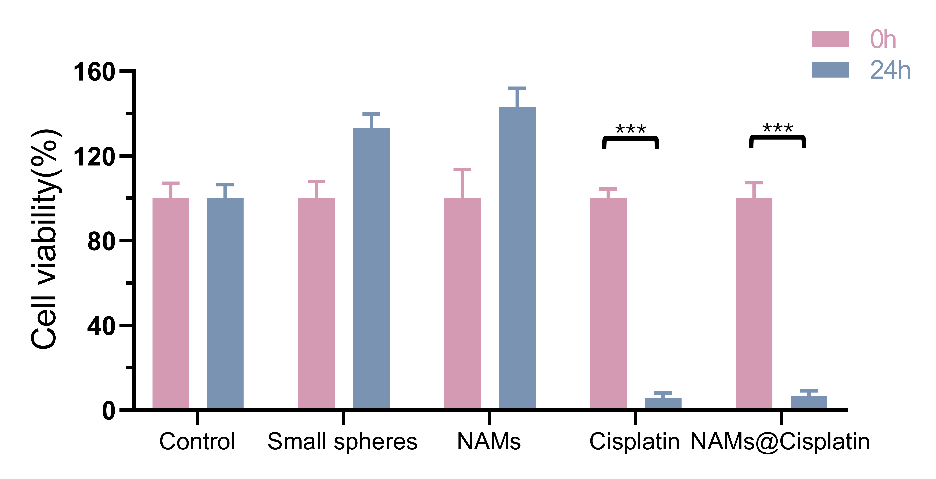


**Supplementary Figure 3.** Cell viability of Hep G2 cells treated with small spheres, NAMs, Free cisplatin and cisplatin-loading NAMs. Data are represented as mean ± standard deviation (n = 6). Significant differences are indicated by asterisks (***p < 0.001).


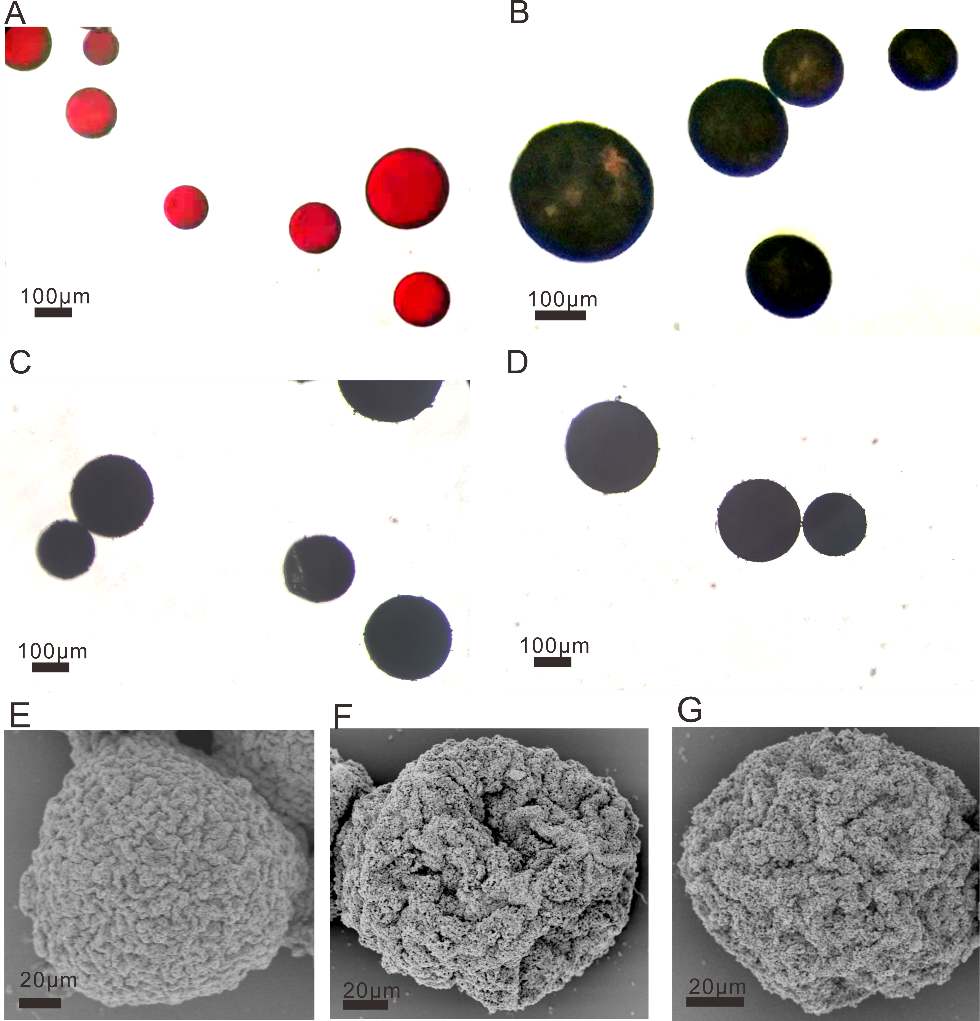


**Supplementary Figure 4.** Characterization of NAMs. Morphologies of NAMs after DOX loading (A) or Cisplatin loading(C), and after DOX release (B) or Cisplatin release (D) under optical microscope. The scale bar is 100 μm for panel A, B, C and D. Representative SEM image of NAMs after DOX loading (E) or Cisplatin loading (F), and after Cisplatin release (G). The scale bar is 20 μm for panel E, F and G.

| Fe Concentration | 0 mM | 0.05 mM | 0.1 mM | 0.2 mM | 0.4 mM |
| --- | --- | --- | --- | --- | --- |
| r2 Value | 6.27±0.22 | 16.76±0.45 | 24.05±0.56 | 31.96±0.61 | 57.18±0.66 |

**Supplementary Table 1.** MRI r2 relaxivity is presented at different Fe concentration. Data expressed as mean ± standard deviation of three independent experiments.
